# Prevention and treatment of COVID‐19 disease by controlled modulation of innate immunity

**DOI:** 10.1002/eji.202048693

**Published:** 2020-06-15

**Authors:** Virgil Schijns, Ed C. Lavelle

**Affiliations:** ^1^ Epitopoietic Research Corporation (ERC) Schaijk The Netherlands; ^2^ Cell Biology and Immunology Wageningen University Wageningen The Netherlands; ^3^ Adjuvant Research Group, School of Biochemistry and Immunology Trinity Biomedical Sciences Institute, Trinity College Dublin Dublin 2 Ireland

**Keywords:** COVID‐19, cytokine, innate immunity, lung, SARS‐CoV‐2

## Abstract

The recent outbreak of coronavirus disease 2019 (COVID‐19), triggered by the severe acute respiratory syndrome coronavirus 2 (SARS‐CoV‐2) poses an enormous threat to global public health and economies. Human coronaviruses normally cause no or mild respiratory disease but in the past two decades, potentially fatal coronavirus infections have emerged, causing respiratory tract illnesses such as pneumonia and bronchitis. These include severe acute respiratory syndrome coronavirus (SARS‐CoV), followed by the Middle East respiratory syndrome coronavirus (MERS‐CoV), and recently the SARS‐CoV‐2 coronavirus outbreak that emerged in Wuhan, China, in December 2019. Currently, most COVID‐19 patients receive traditional supportive care including breathing assistance. To halt the ongoing spread of the pandemic SARS‐CoV‐2 coronavirus and rescue individual patients, established drugs and new therapies are under evaluation. Since it will be some time until a safe and effective vaccine will be available, the immediate priority is to harness innate immunity to accelerate early antiviral immune responses. Second, since excessive inflammation is a major cause of pathology, targeted anti‐inflammatory responses are being evaluated to reduce inflammation‐induced damage to the respiratory tract and cytokine storms. Here, we highlight prominent immunotherapies at various stages of development that aim for augmented anti‐coronavirus immunity and reduction of pathological inflammation.

## Graphical abstract

Innate immune responses to SARS‐CoV‐2 play a decisive role in the outcome of infection. Insufficient or inappropriate responses will lead to increased viremia and cytokine storms while optimal activation or training of innate cells and production of anti‐viral cytokines can control infection and allow a return to homeostasis.

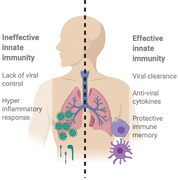

## Innate immune responses

The clinical spectrum of SARS‐CoV‐2 infection involves on the one extreme, asymptomatic cases and patients exhibiting spontaneous recovery, and on the other, a severe acute respiratory syndrome (SARS) characterized by fever, lymphopenia, lung inflammation, immunopathology and potentially death. Coronaviruses attach to their specific cellular receptors via the viral spike protein. The receptor for SARS‐CoV‐2 virus is angiotensin‐converting enzyme 2 (ACE2), a zinc metalloprotease [1].

Diseased lungs of SARS patients displayed increased macrophage and giant‐cell infiltrates as well as hemophagocytosis in the lung, lymphopenia, and white‐pulp splenic atrophy in some cases [2].

A pathogenic role has been proposed for proinflammatory cytokines and chemokines released by stimulated macrophages in the alveoli. Remarkably, despite expression of chemokines such as CXCL10 and CCL2, no IFN‐α/β response was detected in macrophages [3]. SARS‐CoV infection of macrophages in vitro leads to the initiation of viral replication but without producing virus particles. Similarly, in human myeloid‐derived dendritic cells [4] and the epithelial 293 cell line [5], the absence of an IFN‐β response following SARS‐CoV infection was noted. Furthermore, in SARS‐CoV‐1‐infected DCs, low expression of IFNs and IL‐12 was suggested to reflect viral evasion of protective responses [4].

Microarray‐based gene expression profiling of PBMCs from 10 SARS‐CoV‐1 infected patients revealed strong induction of innate inflammatory responses, rather than virus‐specific immune responses [6]. Expression of MHC class I, antiviral cytokine or complement‐mediated cytolysis‐related genes was not significantly increased. These results suggested that the immune response against the SARS‐CoV is different for example from that seen in influenza virus‐infected patients [6].

Patients with SARS experienced a rapid reduction in CD4 and CD8 T cells in peripheral blood during the acute stage of infection that is associated with an adverse outcome [7]. Although SARS‐CoV‐1 can infect and replicate within PBMCs, this replication appears self‐limiting [8] and not responsible for the lymphopenia. Increased production of proinflammatory cytokines (TNF‐α, IL‐6) and chemokines [4] and limited IFN responses [5] suggest viral evasion of host immunity, precipitating SARS pathogenesis, and mortality in susceptible patients. A recent report on patients with severe COVID 19 disease reported higher levels of IL‐2R, IL‐6, IL‐10, and TNF‐α, reduced numbers of CD4^+^ and CD8^+^ T cells and a trend towards lower IFN‐γ expression in CD4^+^ T cells [9]. Circumventing this and promoting a stronger innate antiviral response early after infection may halt viral spread and prevent hyperimmune activation and the respiratory syndrome. Interventions targeting these processes may improve anti‐coronavirus immunity.

## Anti‐SARS biologicals

### SARS‐COV‐2‐specific vaccines

Many companies, universities, and governmental research institutes have embarked on various approaches to develop a prophylactic vaccine specific for SARS‐CoV‐2. A key objective of these approaches is the induction of neutralizing antibodies, to provide protection against coronavirus infection of target cells in the respiratory tract and at other sites of infection. However, the precise immunological correlates of protection against SARS‐CoV‐2 remain to be determined and therefore efforts focused on vaccine‐induced protective CD4^+^ and CD8^+^ T cell responses are also a priority [10]. These accelerated programs are exploiting cutting edge technologies and data on safety and immunogenicity will become available over the coming months. The rapid adoption of parallel approaches with leading technologies from mRNA vaccines to DNA, subunits, and vectored systems offers the greatest opportunity to identify effective vaccines in the coming year(s) [11]. However, while vaccination must be the long‐term solution, it will require at least a year from now before an emerging vaccine(s) is proven to be safe, effective, manufactured, and formally registered.

### Enhancement of non‐specific innate immunity as an antiviral strategy

The tuberculosis vaccine BCG was developed at the beginning of the 20th century and is the most widely used vaccine globally. In addition to its use as a TB vaccine, BCG is also effective clinically for the treatment of bladder cancer [12] where it enhances tumor‐specific CD4^+^ and CD8^+^ T cell responses. According to a number of studies in animal models, prophylactic vaccination with BCG also increases resistance to infections by viruses, including influenza virus and herpes simplex (HSV) type 2, with less viral tissue damage and reduced viral replication [13, 14, 15]. However, no protection was seen when it was tested therapeutically after infection. In another mouse study, immunization with BCG was not protective against an H7N9 influenza infection [16]. The study of Mukherjee and co‐workers found that delivery of low dose BCG (TICE strain) was protective against (PR8) (H1N1) influenza infection only after intranasal (i.n.) but not subcutaneous administration [15]. Similarly, nasally delivered BCG was more protective than intraperitoneal injection against influenza A (H1N1) PR8 challenge [14]. This suggests that the route of BCG administration might be important in the case of protection against respiratory influenza infection and may highlight a requirement to specifically boost mucosal responses for optimal protective immunity.

While systemic injection or i.n. administration of BCG will not itself induce SARS‐CoV‐2‐specific adaptive immunity, its application aims to boost innate immunity to bolster protection against the virus. In the current crisis, such enhancement of innate immunity may aid rapid recognition of SARS‐CoV‐2 virus and reduce replication and spreading [17]. Recently, a Dutch trial commenced in 1000 health care workers who will either receive the BCG vaccine, or a placebo to monitor the efficacy of BCG immunization against SARS‐CoV‐2. In a randomized clinical trial, Arts et al., recently showed that vaccination with BCG correlates with protection against experimental viral infection with a weakened form of the yellow fever virus, which is used as a vaccine. Vaccination with BCG 1 month prior to yellow fever virus vaccine resulted in significantly lower viremia compared to subjects who had received the placebo. This protective effect correlated with upregulation of IL‐1ß production [18]. Similarly, in at least one Phase III study in Germany, researchers from Vakzine Project Management (VPM) and the Serum Institute of India, will investigate whether vaccination with the TB vaccine candidate, VPM1002, a genetically modified version of BCG (developed by scientists at the Max Planck Institute for Infection Biology), is effective against SARS‐CoV‐2 infection.

### Non‐specific immunostimulatory adjuvants

In addition to BCG, live viral vaccines and other innate immune stimuli have the potential to promote “trained immunity” and beneficially boost protective antiviral immunity. In particular beta (β)‐glucans [19] but possibly also other formulated vaccine adjuvants, nanoparticles, emulsions, natural, or synthetic ligands of innate immune cells receptors including agonists for Toll‐like receptors, NOD‐like receptor, RIG‐I‐like receptor, and C‐type lectin receptors [20]. Hence, a variety of microbial components may enhance innate immunity and thereby potentially increase resistance to viral infection. The FDA has approved an investigation into the efficacy of an inhalational PUL‐042 solution that contains TLR agonists that can activate lung epithelial cells as a means to reduce the severity of COVID‐19 (NCT04312997, NCT04313023). Earlier work demonstrated the potential of muramyl dipeptide to promote non‐specific antiviral immunity [21, 22]. Nonspecific modulation of cytokine production is well known from the field of vaccine adjuvants even if it has not been widely translated [23]. An innate training inducer that could promote type 1 IFNs may be particularly suitable in this setting.

### Therapeutic potential of IFNs

During most viral infections, infected cells secrete high concentrations of type 1 IFNs (IFN‐α and IFN‐β). These broadly acting immunomodulatory IFNs induce an antiviral state in neighboring cells, thereby preventing viral spread by increasing the resistance of uninfected cells toward the virus. In addition, IFNs can activate NK cell cytotoxicity toward virus‐infected cells. IFN‐α/β further contributes to driving the adaptive‐immune response towards beneficial antiviral Th type 1 (Th1) responses, via stimulation of IFN‐γ expression and DC programming. The poor induction of IFN‐β, has been suggested as a contributing factor to SARS pathogenesis [3].

Type 1 IFNs are used therapeutically in other contexts (such as hepatitis C and cancer therapies) so the dosing schedule, pharma kinetic profile, efficacy against other viruses and potential side effects of IFN‐α/β therapies are all well known. Hence, various commercial brands of recombinant type I IFNs are readily available. They may be especially useful for prophylactic interventions or at the early onset of first symptoms. A study in cynomolgus macaques found that both prophylactic administration and postexposure delivery of pegylated IFN‐α was clinically beneficial against SARS‐CoV‐1 coronavirus infection [24], confirming studies in other animal models [25]. Prophylactic administration reduced virus replication and excretion, viral antigen expression in pneumocytes and pulmonary damage. A study in humans indicated that inhaled IFN‐β was a potential treatment for virus‐induced deteriorations of asthma. IFN‐β enhanced innate immunity locally within the lungs, thereby compensating for the IFN‐β deficiency demonstrated in the epithelium of patients with moderate‐severe asthma [26]. Since type 1 IFNs can enhance IL‐10 production by T cells, type 1 IFN delivery may serve a dual function in enhancing antiviral immunity while blunting the damaging inflammatory response.

As well as direct administration of interferons, there is scope to use immunostimulatory molecules than can induce IFN secretion by cells, particularly in the respiratory tract. There are range of innate immune sensors that can promote strong IFN responses including endosomal toll like receptors, the RNA helicases RIG‐I and MDA5 and the DNA sensor cGAS. While SARS coronaviruses can evade immune responses by blocking the induction of interferons and reducing IFN‐driven responses, evidence from SARS‐CoV‐1 coronavirus suggests that type 1 IFN derived from plasmacytoid DCs can control infection [27]. Furthermore, although SARS CoV‐1 did not induce type 1 IFNs in fibroblasts, SARS CoV viral infection did not block the induction of an IFN response by treatment of cells with poly IC or Sendai virus [28]. Therefore, there may be scope to test agonists of a range of innate sensors to assess whether it is possible to circumvent SARS‐CoV‐2 evasion of type 1 IFNs and provide a degree of protection in the respiratory tract.

In addition to IFNs, other cytokines may have potential to improve outcomes as a preventive approach shortly before infection or during postexposure prophylaxis. For example, IL‐22 has been shown to promote host defense against viral (e.g., influenza) and bacterial (super) infection, and may represent a strategy to improve epithelial barrier function [29, 30]

When applying immune response enhancing recombinant cytokines as a potential anti‐SARS‐CoV‐2 agent, the timing of administration and its duration are likely to be critical. Shortly before exposure to (potential) infection the presence of sufficient exogenous type 1 IFN is expected to beneficially initiate an antiviral inflammatory response and an antiviral state [24, 25], while prolonged IFN therapy or the presence of IFN too late after viral exposure may enhance detrimental immunopathological effects causing lung tissue damage [31, 32].The potential of type 1 IFN to enhance ACE2 expression must also be considered [33, 34].

## Antibodies for passive protection

SARS‐CoV‐2 virus‐specific polyclonal hyperimmune antibodies derived from plasma of recovered patients may neutralize the virus and protect from COVID‐19 disease. In several countries, blood donors who recovered from SARS‐CoV‐2 are currently recruited to donate plasma for hyperimmune serum production. A recent publication reported improvement in the clinical status of five critically ill COVID 19 patients following administration of convalescent plasma containing virus neutralizing antibodies [35].

While this is a critical proof of concept for the ability of antibodies from surviving patients to neutralize the virus, it is not a feasible approach for broader treatment of infected populations. Several companies have identified monoclonal antibodies that can bind to SARS‐CoV‐2 spike (S) protein. Intravenous administration of two MERS coronavirus S‐protein specific monoclonal antibodies (REGN3048 and REGN3051) in a common marmoset model of MERS resulted in effective viral neutralization with reduced viral loads in the lungs [36]. However, while this was effective in a prophylactic setting, it showed limited benefit when applied following infection [37, 38]. Importantly, in SARS‐CoV‐1 infected macaques, immunization with a modified vaccinia Ankara (MVA) virus encoding full‐length SARS‐CoV S glycoprotein, induced anti‐viral antibodies targeting the SARS‐CoV‐spike protein that may cause severe acute lung injury [39]. Hence, the quality and the specificity of the anti‐SARS‐CoV‐2‐specific antibodies induced by immunization [10] or administered passively [40] may affect their capacity for protection or exacerbation of disease.

Apart from direct virion neutralization and prevention of viral entry into target cells, antiviral antibodies may also enhance innate immune cell effector functions such as antibody dependent cellular cytotoxicity and complement dependent cytotoxicity by engaging Fc receptors [41, 42]. Such activation of innate immune cells including NK cells, monocytes, and macrophages by antibody‐bound target cells may contribute to the destruction of virion‐producing infected cells.

## Immunomodulatory Anti‐SARS Drugs

### Chloroquines

Chloroquine (CQ) and hydrochloroquine (HCQ) have been used for the treatment of malaria and amebiasis. Hydroxychloroquine (HCQ) sulfate is a less toxic derivative of CQ, and based on its anti‐inflammatory properties, has been used to treat autoimmune diseases, such as systemic lupus erythematosus and rheumatoid arthritis. CQs were shown to dampen innate immune responses by suppressing endosomal TLR activation as a result of direct binding to nucleic acids, thereby inhibiting TLR ligand interaction [43] and reducing TLR (7, 8, and 9) signaling and cytokine production. In addition, the molecules can interfere with lysosomal activity and autophagy, impair antigen presentation, and reduce CD154 expression in T cells [44]. Keyaerts and co‐workers showed that chloroquine inhibits SARS‐CoV‐1 replication in vitro [45]. Recently, Liu and co‐workers showed that HCQ also inhibit SARS‐CoV‐2 infection in vitro [46]. Since February 23, 2020, several clinical trials have commenced to evaluate HCQ for the treatment of COVID‐19 [47]. While it was reported that chloroquine phosphate demonstrated efficacy in infected patients [48] and the US Food and Drug Administration (FDA) provided emergency use authorization for chloroquine (hydroxychloroquine/Plaquenil) in specific hospitalized groups, a recent editorial concluded based on the current evidence that use of these drugs is premature and potentially harmful [49].

### Azithromycin

The macrolide antibiotic Azithromycin has been demonstrated to influence immune parameters, such as increasing antiviral IFN expression in both healthy and in virus‐infected cells, and to exert antiviral and anti‐inflammatory actions, thereby reducing exacerbations in chronic obstructive pulmonary disease (COPD) [50, 51]. It was claimed that addition of Azithromycin to chloroquine improved therapeutic effects in a non‐randomized clinical trial [52].

### Targeting excessive SARS‐CoV‐2 induced inflammatory responses

Immune responses to lung infections in general, and SARS‐CoV‐2 in particular, must be carefully regulated in order to achieve prompt and effective viral eradication while maintaining gas exchange and organ function. In susceptible patients, an acute respiratory distress syndrome (ARDS), typically observed for the SARS‐CoV‐2 virus infection is associated with alveolar flooding, cytokine storms, and interstitial inflammation [9]. This results in respiratory failure and the need for artificial ventilation. Cytokine storms can also result in organ failure and death. Hence, there is scope for immunomodulatory strategies to inhibit or blunt excessive activation of the local inflammatory response.

IL‐6 is a widely used biomarker of inflammation. Elevated circulating IL‐6 is associated with higher mortality in patients with community acquired pneumonia. IL‐6 is also implicated in driving an excessive inflammatory response in the lungs of COVID‐19 patients and a number of strategies are being evaluated to blunt IL‐6 driven inflammation. For example, antibodies targeting IL‐6 are approved for the treatment of rheumatoid arthritis and a number of IL‐6 targeting monoclonal antibodies are under evaluation as treatments for severe COVID‐19 disease. For example, the anti‐IL‐6 monoclonal, sarilumab (Kevzara®) is being evaluated in a phase 2/3 clinical trial (Regeneron/Sanofi) in patients with severe SARS‐CoV‐2 virus infection. In addition to targeting the cytokine, the IL‐6 receptor is a potential target to reduce acute inflammatory responses. The human anti‐IL‐6 receptor antibody, TZLS‐501 (Tiziana Life Sciences) may prevent lung damage and elevated circulating levels of IL‐6 and is being tested for the treatment of COVID‐19. The IL‐6 receptor antagonist, tocilizumab (Actemra®, Roche) was approved in China for the treatment of severe complications related to coronavirus infection and the FDA recently approved a phase 3 trial of Actemra in severely ill COVID‐19 patients, who have been hospitalized with pneumonia. IL‐6 is not the only inflammatory cytokine being investigated as a means to reduce pathological inflammation and cytokine storms. A neutralizing antibody targeting GM‐CSF (TJM2, Mab Biopharma) is being evaluated as a treatment for cytokine storm in patients suffering from a severe case of coronavirus infection and therapies to inhibit IL‐1 and block inflammasome activation are being evaluated.

## Concluding remarks

While a SARS‐CoV‐2 vaccine will take some time to develop, there are a range of other immune interventions that are being tested and others that could be conceived as a means to boost protective innate immunity and reduce damaging inflammatory responses. At advanced disease stages, blocking IL‐6 appears a promising approach to reduce virus‐induced inflammation and cytokine storms, but other anti‐inflammatory modalities targeting detrimental cytokines or chemokines can also be considered. As a result of the outstanding progress in clinical application of biologics for inflammatory diseases over the past decade, a number of anti‐cytokine tools are available that can be tested.

Over recent years, the process of inflammaging has been highlighted and the lungs of elderly individuals are characterized by chronic low‐grade inflammation [53]. In the case of SARS‐CoV, it was shown that innate host responses were exacerbated in aged non‐human primates [54]. Furthermore, rapid upregulation of ARDS‐associated cytokines was lethal in an aged mouse model of SARS‐CoV‐1 infection [55]. Thus, there may be potential to address underlying defects associated with inflammaging to overcome detrimental age‐related innate and adaptive immune responses [56]. These could include addressing microbial dysbiosis, with, for example microbiome targeting pre‐ or probiotics in certain risk groups, since intestinal microbiome dysbiosis is known to trigger inflammation and affect immune responsiveness [57, 58]. Hence, aiming for microbiome balance may prevent dangerous immune over‐reactions that damage the lungs and other vital organs.

**Figure 1:**
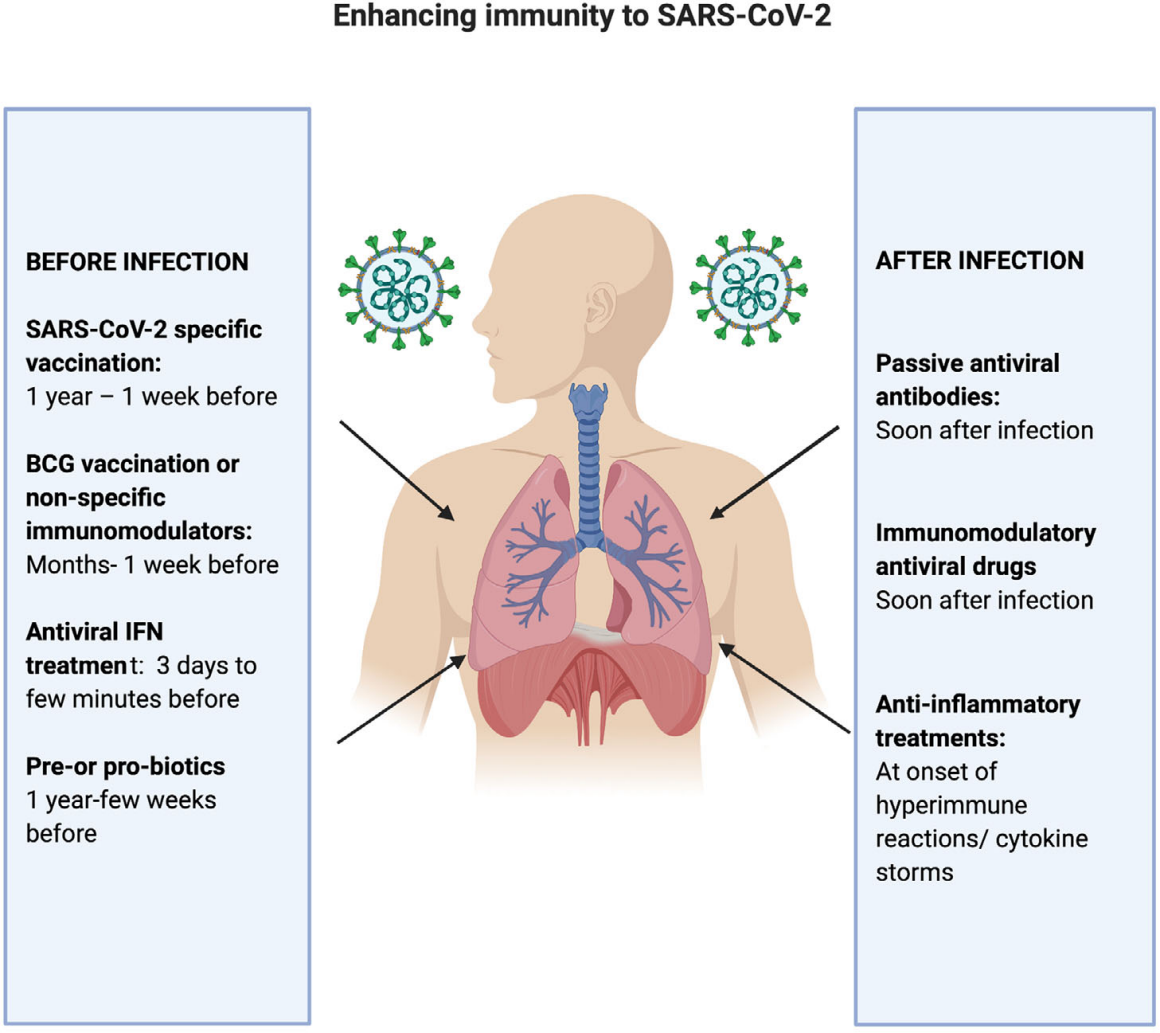
**Immunological interventions to enhance responses to Covid‐19 disease**. On the left panel are interventions that can be used prior to infection including specific vaccines, IFNs, agents to train or boost innate immunity or agents to address dysbiosis. On the right are interventions following infection that include passive transfer of convalescent antiviral sera or virus‐specific monoclonal antibodies, immunomodulatory antiviral drugs, and anti‐inflammatory treatments, particularly inhibitors of deleterious cytokines.

As highlighted above, before a suitable SARS‐CoV‐2 virus‐specific vaccine is developed, there are a number of options to boost antiviral immune responses by delivering IFNs or IFN‐inducing agents or by nonspecific boosting of immunity with innate factors or vaccines. These immunomodulatory approaches could be introduced before or shortly after viral exposure and combined with antiviral therapies for example antiviral nucleotide analogues such as remdesivir. Therapeutic options include passive antiviral antibody transfer. However, in all cases it is essential not to exacerbate damaging inflammatory responses and therefore the stage of infection when the intervention is made is critical (Fig. 1). While some cytokines such as IL‐1 can be highly inflammatory they may also contribute to protective T cell responses [59] and appropriate bystander T cell activation could contribute to antiviral response. A key aspect to address is the optimal type of mucosal immunity to mediate local protection. Characterizing the optimal class/subclass of neutralizing antibodies in sera and the lungs and the nature of protective CD4 and CD8 immunity can inform therapeutic approaches and also vaccine design.

## Conflict of interest

The authors declare no commercial or financial conflict of interest.

**Abbreviations**
ACE2: angiotensin‐converting enzyme 2
ARDS: acute respiratory distress syndrome
CQ: chloroquine
HCQ: hydrochloroquine
HSV: herpes simplex virus
SARS: severe acute respiratory syndrome
